# Effect of inotropic agents on oxygenation and cerebral perfusion in acute brain injury

**DOI:** 10.3389/fneur.2022.963562

**Published:** 2022-07-19

**Authors:** Giacomo Coppalini, Elie Duvigneaud, Alberto Diosdado, Ernesto Migliorino, Sophie Schuind, Jacques Creteur, Fabio Silvio Taccone, Elisa Gouvêa Bogossian

**Affiliations:** ^1^Department of Intensive Care, Erasme Hospital, Université Libre de Bruxelles, Route de Lennik, Brussels, Belgium; ^2^Department of Neurosurgery, Erasme Hospital, Université Libre de Bruxelles, Route de Lennik, Brussels, Belgium

**Keywords:** hemodynamics, acute brain injury, cerebral blood flow, brain oxygenation, inotropic agents

## Abstract

**Introduction:**

Tissue hypoxia and insufficient energy delivery is one of the mechanisms behind the occurrence of several complications in acute brain injured patients. Several interventions can improve cerebral oxygenation; however, the effects of inotropic agents remain poorly characterized.

**Methods:**

Retrospective analysis including patients suffering from acute brain injury and monitored with brain oxygen pressure (PbtO_2_) catheter, in whom inotropic agents were administered according to the decision of the treating physician's decision; PbtO_2_ values were collected before, 1 and 2 h after the initiation of therapy from the patient data monitoring system. PbtO_2_ “responders” were patients with a relative increase in PbtO_2_ from baseline values of at least 20%.

**Results:**

A total of 35 patients were included in this study. Most of them (31/35, 89%) suffered from non-traumatic subarachnoid hemorrhage (SAH). Compared with baseline values [20 (14–24) mmHg], PbtO_2_ did not significantly increase over time [19 (15–25) mmHg at 1 h and 19 (17–25) mmHg at 2 h, respectively; *p* = 0.052]. A total of 12/35 (34%) patients were PbtO_2_ “responders,” in particular if low PbtO_2_ was observed at baseline. A PbtO_2_ of 17 mmHg at baseline had a sensibility of 84% and a specificity of 91% to predict a PbtO_2_ responder. A significant direct correlation between changes in PbtO_2_ and cardiac output [r = 0.496 (95% CI 0.122 to 0.746), *p* = 0.01; *n* = 25] and a significant negative correlation between changes in PbtO_2_ and cerebral perfusion pressure [*r* = −0.389 (95% CI −0.681 to −0.010), *p* = 0.05] were observed.

**Conclusions:**

In this study, inotropic administration significantly increased brain oxygenation in one third of brain injured patients, especially when tissue hypoxia was present at baseline. Future studies should highlight the role of inotropic agents in the management of tissue hypoxia in this setting.

## Introduction

Cerebral hypoxia is a major cause of secondary brain injury that can be observed in a relevant proportion of patients with acute brain injury (ABI), including patients with traumatic brain injury (TBI), subarachnoid hemorrhage (SAH) and intracerebral hemorrhage (ICH) (1–5). Indeed, low brain tissue oxygenation (PbtO_2_), which can be measured at bedside using an intraparenchymal probe placed into the “at-risk” areas, has been associated with cerebral anaerobic metabolism, as well as with an increased risk of mortality and poor functional outcome in these patients (6–12).

Low PbtO_2_ values have been observed in different conditions, such as reduced cerebral blood flow (CBF) and/or cerebral perfusion pressure (CPP), intracranial hypertension (IH), hypoxemia, anemia, altered microcirculation or excessive cellular metabolism (13). As such, several therapeutic strategies have been developed to optimize brain oxygenation into a complex and protocolized algorithm, which encompasses vasopressors (i.e., to increase CPP), treatments of IH (i.e., osmotic therapy), red blood cells transfusions or increased inspired oxygen fraction (FiO_2_), temperature control and sedation (i.e., to decrease cerebral oxygen consumption) (14–18).

In case of reduced CBF, one of the most commonly used intervention is the increase in CPP (i.e., induced hypertension) with normovolemia; in patients suffering from SAH, this intervention can increase regional perfusion in the presence of cerebral vasospasm and neurological deterioration (19). Among TBI patients, this strategy can increase tissue oxygenation and reduce intracranial pressure, if cerebral autoregulation is preserved (14, 15, 20). In patients with ICH, although it is generally recommended to control blood pressure to avoid hematoma expansion (21), increased CPP was associated with higher PbtO_2_ values in those patients with poor clinical condition at presentation (18, 22).

Interestingly, the effects of inotropes, such as dobutamine (a β_1_ agonist) and milrinone (a phosphodiesterase-3 inhibitor) on brain oxygenation remains unknown. These drugs have been proposed as “second-line” agents to improve CBF and CPP in acute brain injury patients (19, 23), or as a support therapy in those suffering from neurogenic heart failure (24–26). Therefore, the aim of this study was to assess the effect of inotropic agents on PbtO_2_ after an acute brain injury.

## Methods

### Study design

This is a single center retrospective analysis of prospectively collected data conducted at Erasme University Hospital, Brussels, Belgium. All adults patients (>18 years old) admitted to the Intensive Care Unit of Erasme hospital from January 2015 to February 2022, due to acute brain injury (SAH, TBI or ICH), who survived at least 24 h were eligible for inclusion. The study protocol was approved by the local ethics committees (SRB2022006). Data collection and analyses were carried out in accordance with relevant scientific and ethical guidelines and regulations.

### Inclusion criteria and exclusion criteria

We included eligible patients who were monitored with intracranial pressure (ICP) and PbtO_2_ and who received inotropic treatment (i.e., either by dobutamine or milrinone) during monitoring, according to the treating physician's decision. We excluded patients who had malfunctioning/unreliable PbtO_2_ readings or unavailable data.

### Patient's management

Current guidelines for the management of TBI (20), SAH (27), and ICH (21) were implemented in clinical practice; invasive multimodal neuro-monitoring, including PbtO_2_, was included according to a recent consensus (28) and considered as “standard of care” for acute brain injury patients with a Glasgow Coma Score (GCS) <9 and requiring intracranial pressure (ICP) monitoring. PbtO_2_ catheter was preferably placed into the “at risk” area, i.e., close to the injured/contused area in TBI and ICH patients and in the region at risk (vascular territory of the artery harboring the aneurysm) or with demonstrated initial or delayed hypoperfusion on brain imaging for SAH.

### Data collection

Physiological variables as well as ICP and PbtO_2_, were measured in real-time and collected prospectively. Cerebral perfusion pressure (CPP) was calculated as the difference between mean arterial pressure (MAP) and ICP; MAP was zeroed at level of left atrium. Intracranial hypertension was defined by the observation of ICP values above 20 mmHg for at least 5 min at any time. Brain tissue hypoxia was defined as a PbtO_2_ <20 mmHg. At baseline (immediately before the start of inotropic therapy), 1 and 2 h after the start of inotropes, the 20-min mean value of the following variables was collected: PbtO_2_, MAP, heart rate (HR), ICP, CPP, body temperature, arterial oxygen saturation (SaO_2_), central venous oxygen saturation (SvcO_2_), cardiac output (CO), pH, PaCO_2_, PaO_2_, blood lactate and blood glucose levels. Vasopressors and inotropic doses were also recorded. We also calculated the ΔPbtO_2_ defined as the difference between PbtO_2_ values at 2 h and at baseline; similarly, the ΔCO, ΔICP, ΔCPP, and ΔPaCO_2_ were also computed.

We also recorded demographics and the presence of comorbidities, the Sequential Organ Failure Assessment (SOFA) (29) and the Acute Physiology and Chronic Health Evaluation (APACHE) II scores (30) on admission, as well as the GCS score (31) on admission, the use of mechanical ventilation, vasopressor and renal replacement therapy (RRT). The occurrence of neurological complications, such as epilepsy, hydrocephalus, rebleeding, delayed cerebral ischemia and intracranial hypertension, was collected.

Hospital mortality and the Glasgow Outcome Scale (GOS) (32) at 6 months was also reported, either collected from the medical charts or via the general practitioner. Unfavorable neurological outcome was defined as GOS of 1–3.

### Outcomes

The primary outcome of the study was the changes in PbtO_2_ within 2 h after the initiation of inotropic therapy. PbtO_2_ increase was calculated as the difference between the highest mean PbtO_2_ values, measured at 1 or 2 h after the initiation of inotropic therapy, and the baseline PbtO_2_ value; PbtO_2_ “decrease” was identified as a difference value <0, “stable” PbtO_2_ as a difference = 0 and “increase” as a difference >0. A “significant” increase was defined as > 20% from the baseline. Patients that experienced a significant increase were defined as “responders.” Secondary outcomes included the identification of factors associated with a significant PbtO_2_ increase.

### Statistical analysis

Descriptive statistics were computed for all variables. Normality was assessed using the Kolmogorov-Smirnov test. Non-gaussian continuous variables were described as median and interquartile range [IQRs] and compared using Mann-Whitney test (independent variables) or Friedman/Wilcoxon test (repeated measures of related variables); normally distributed variables were expressed as mean (±SD) and compared using Student *t*-test. Categorical variables were described as proportions (%) and compared using Chi square or Fisher's exact test. A logistic regression of physiological variables at baseline was conducted to assess factors associated with a significant PbtO_2_ increase after inotropic therapy. The independence of errors, presence of multicollinearity and of influential outlier assumptions were checked; none were violated. The area under the receiver operator characteristic (AUROC) was computed to identify the optimal cut-off value (i.e., best sensitivity and specificity—Youden's index) of baseline PbtO_2_, baseline ICP and baseline CPP to predict a significant increase of PbtO_2_ after inotrope infusion. We performed a correlation analysis using Spearman's test between ΔPbtO_2_ and ΔCO, ΔICP, ΔCPP, and ΔPaCO_2_. The R coefficient and the 95% confidence interval were computed for all correlations. We also performed a linear regression analysis with ΔPbtO_2_ as the dependent variable and ΔCO, ΔICP, ΔCPP, and ΔPaCO_2_ as covariates. All statistical analyses were performed using SPSS 27.0 for MacIntosh. A *p*-value <0.05 was considered significant.

## Results

### Characteristics of the study participants

On a total of 157 patients monitored over the study period, 35 patients were eligible for our study. The characteristics of the study population are shown in Supplementary Table 1. Most patients presented with SAH (31/35, 89%); SAH patients had predominantly a poor grade status at presentation [WFNS 4 or 5: 23/31(74%)] and a high radiological scale [mFisher 3 or 4: 29 (94%)]. The median age was 55 (46–62) years, with a slight predominance of male patients (18/35, 51%). The median GCS on admission was 9 (3–13) and the most common complication was the occurrence of intracranial hypertension. Ninety-one percent of patients experienced at least one episode of brain tissue hypoxia over the ICU stay. All patients received dobutamine as the studied inotrope except one patient who received milrinone. Overall, in-hospital mortality was 57%; 71% of patients had an unfavorable neurological outcome at 6 months.

### Inotropic agents and brain oxygenation

An increase in PbtO_2_ was observed in 22/35 patients (63%); of those, 12 (55% of patients with PbtO_2_ increase and 34% of the whole cohort) experienced a significant PbtO_2_ increase from baseline (responders). In 4 patients, PbtO_2_ did not change while it decreased in 9 patients (26%).

**Figure 1:**
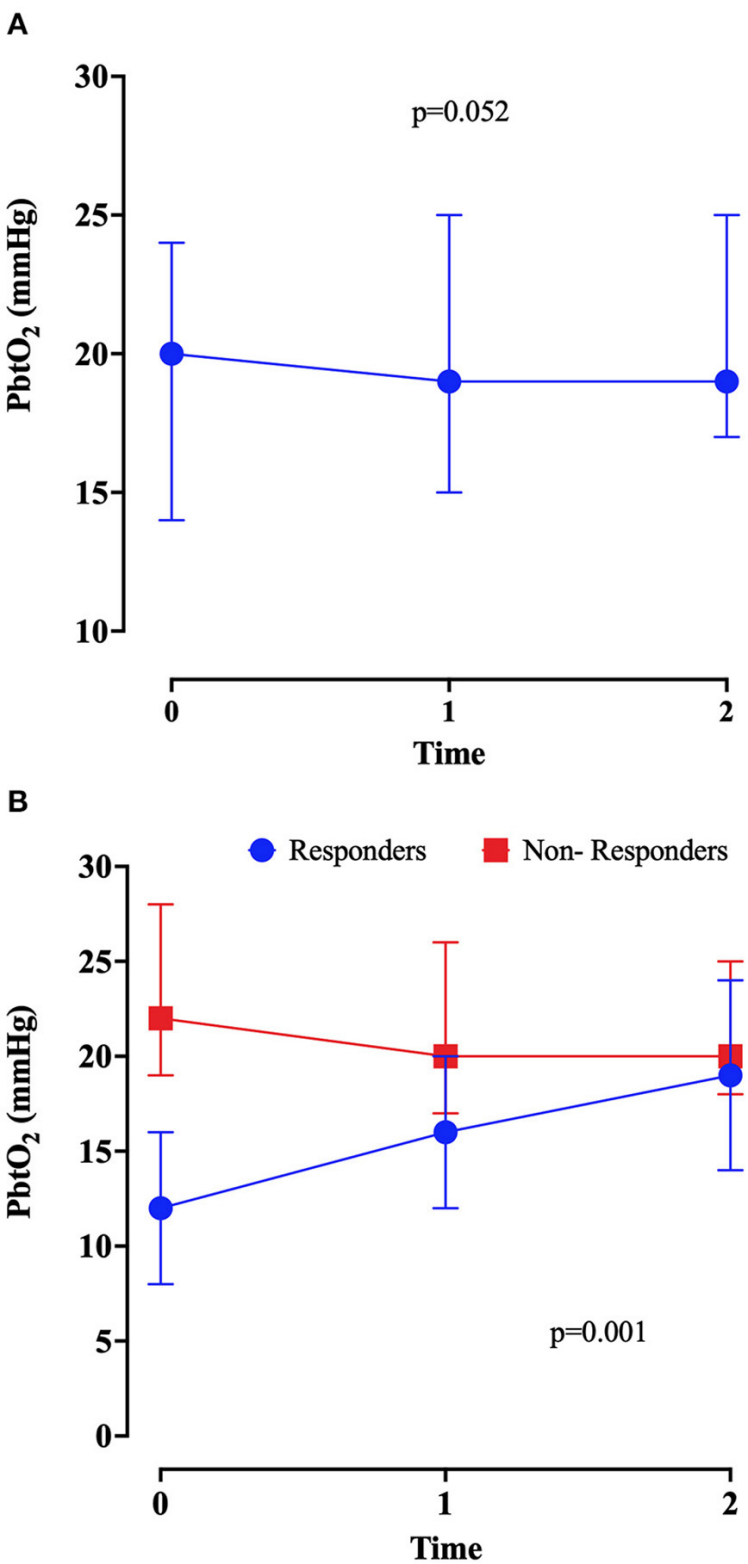
Brain tissue oxygenation (PbtO_2_) values at baseline (before initiation of inotropes—T0), at 1 (T1) and 2 (T2) hours after the introduction of inotropic continuous infusion in the overall population **(A)** and in responders and non-responders **(B)**. *P*-value was calculated by Friedmann test.

There was a non-significant change in PbtO_2_ after the initiation of the inotropic agents, as shown in Table 1 and Figure 1 (*p* = 0.052). Also, a significant increase in heart rate and in cardiac output was observed after inotropic agents' administration; no changes in CPP values were observed. The doses of dobutamine are reported in Table 2; milrinone was given at 0.375 mcg/kg^*^min.

**Table 1:** Changes in main variables from baseline values after inotropic agents administration.

***Variables***	**Baseline**	**1-hour**	**2-hour**	**Value**
PbtO_2_, mmHg	20 (14–24)	19 (15–25)	19 (17–25)	0.052
Hb, g/dL	11 (9.7–12.6)	10.4 (9.4–12.3)	10.3 (9.3)	0.72
MAP, mmHg	118 (106–132)	120 (109–132)	119 (98–131)	0.16
CPP, mmHg	109 (93–118)	110 (98–120)	109 (88–120)	0.38
ICP, mmHg	13 (7–16)	12 (8–19)	10 (7–17)	0.16
HR, bpm	86 (75–110)	93 (80–107)	104 (86–109.6)	0.01
Temperature, °C	37.0 (35.8–37.5)	36.9 (35.7–37.3)	37 (35.6–37.4)	0.80
pH	7.41 (7.35–7.46)	7.43 (7.36–7.45)	7.42 (7.37–7.44)	0.06
PaO_2_, mmHg	110 (92–148)	108 (88–129)	112 (94–135)	0.13
PaCO_2_, mmHg	42 (39–47)	43 (37–46)	43 (39–49)	0.02
Lactate, mmol/L	1.1 (0.8–1.4)	1.1 (0.8–1.7)	1.2 (0.9–1.8)	0.23
SaO_2_, %	99 (98–99)	99 (98–99)	99 (98–99)	0.84
Glucose, mg/dL	135 (119–174)	154 (117–188)	163 (133–199)	0.04
SvO_2_, %*	79.7 (75.3–83)	78.4 (74–82)	83.9 (83.3–87.6)	0.37
CO, L/min*	6.1 (5.0–8.0)	7 (5.9–8.4)	7.45 (6.6–8.6)	0.001
Norepinephrine, mcg/kg/min	0.4 (0.14–1.4)	0.43 (0.17–1.53)	0.36 (0.16–1.59)	0.29
Dobutamine, mcg/kg/min	–	4 (3–5)	5 (3–5)	0.001

*Values are presented as median (IQR). Values in bold represent statistical significance*.

PbtO_2_, brain tissue oxygenation partial pressure; Hb, hemoglobin; MAP, mean arterial pressure; CPP, cerebral perfusion pressure mmHg; ICP, intracranial pressure; HR, heart rate; bpm, beats per minute. SaO_2_, Oxygen saturation; PaO_2_, Oxygen partial pressure; PaCO_2_, Carbon dioxide partial pressure. ^*^n = 25 (n = 7 for responders and n = 18 for non-responders).

**Table 2:** Logistic regression multivariable analysis to predict a significant PbtO_2_ increase after inotropic treatment.

	**Univariable OR (95% CI)**	**Multivariable OR (95% CI)**	***p*** **-value**
Baseline PbtO_2_, mmHg	0.79 (0.66–0.93)	0.82 (0.68–0.98)	0.03
Baseline ICP, mmHg	1.19 (1.02–1.39)	1.20 (0.97–1.48)	0.10
Baseline CPP, mmHg	0.96 (0.92–0.99)	0.97 (0.92–1.02)	0.25

*PbtO_2_, brain tissue oxygen partial pressure; ICP, intracranial pressure; CPP, cerebral perfusion pressure; OR, odds ratio; CI, confidence interval*.

There was a significant direct correlation between ΔPbtO_2_ and ΔCO [*r* = 0.496 (95% CI 0.122 to 0.746), *p* = 0.01; *n* = 25] and a significant negative correlation between ΔPbtO_2_ and ΔCPP [*r* = −0.389 (95% CI −0.681 to −0.010), *p* = 0.05], as shown in Supplementary Figure 1. There was a weak non-significant correlation between ΔPbtO_2_ and ΔICP [*r* = 0.26 (95% CI −0.151 to 0.597)]. Linear regression analysis described the association of ΔPbtO_2_ and ΔCPP [beta coefficient −0.274 (95% CI −0.130 to 0.041)], adjusted for ΔICP [beta coefficient 0.018 (95% CI −0.139 to 0.148)], ΔPaCO_2_ [beta coefficient −0.038 (95% CI −0.026 to 0.021)] and ΔCO [beta coefficient 0.371 (95% CI −0.054 to 1.306)].

### PbtO_2_ responders

Main differences in collected variables at baseline between responders and non-responders are presented in Supplementary Table 2. Responders had more frequently seizures and rebleeding than non-responders; also, PbtO_2_ and CPP were significantly lower, while ICP higher, than non-responders at baseline. No significant changes in measured variables over time were observed between groups, except a higher requirement in norepinephrine in the “responders” group when compared to the others (Supplementary Table 3). No statistically significant difference in hospital mortality and neurological outcome at 6 months between responders and non-responders was observed (Supplementary Table 2); no multivariable analysis was performed due to the limited sample size. Among survivors (*n* = 15), responders [GOS 4 (4, 5)] had numerically higher GOS at 6 months than non-responders [GOS 4 (3, 4)—*p* = 0.30]. No differences in mFisher and WFNS score on admission between responders and no responders were observed (Supplementary Table 2).

**Figure 2:**
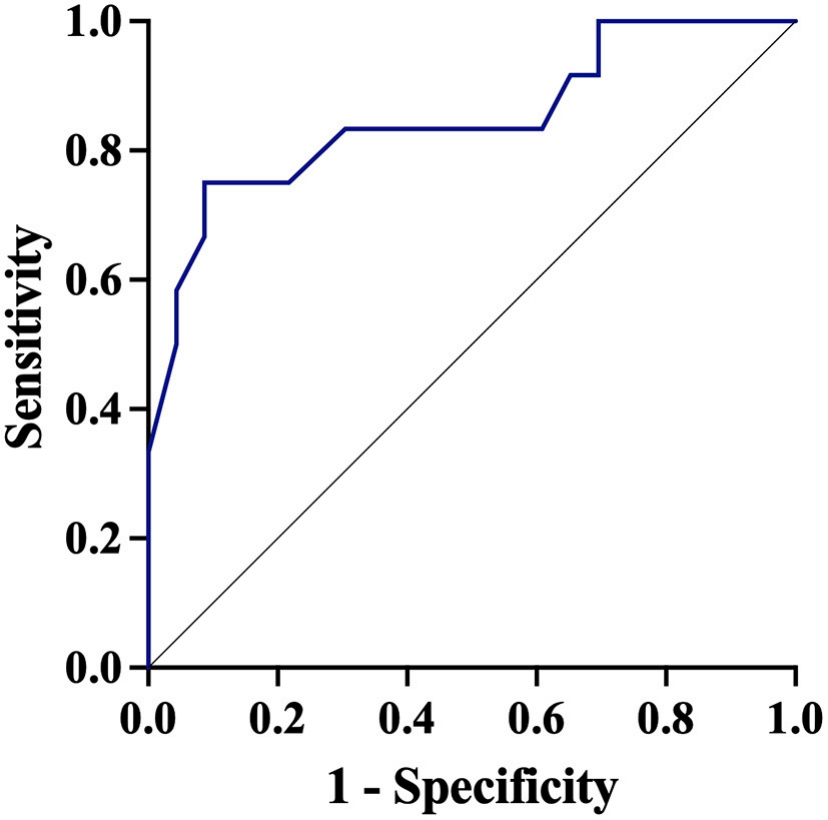
Receiver Operator Curve (ROC) of baseline brain oxygen pressure (PbtO_2_) to predict a significant PbtO_2_ increased after therapy.

In a multivariable model, lower baseline PbtO_2_ was associated with higher probability of a significant PbtO_2_ increase after inotropic treatment (Table 2). The AUROC for baseline PbtO_2_ (Figure 2) to predict a significant PbtO_2_ increase was 0.85 (95% CIs 0.70–1.00). The baseline PbtO_2_ cut-off that better discriminated between responders and non-responders was 17 mmHg, with a sensibility of 84% and a specificity of 91% to predict a significant PbtO_2_ increase. The AUROC for CPP and ICP and response to predict a significant PbtO_2_ increase were 0.72 (95% CIs 0.50–0.94—best cut-off of 99 mm Hg) and 0.76 (95% CIs 0.60–0.92—best cut-off of 14 mmHg), respectively. In SAH patients, a multivariable model adjusted for poor grade on admission (WFNS 4 or 5) and mFisher, lower baseline PbtO_2_ was associated with a higher chance of response to inotropes (Supplementary Table 4).

## Discussion

In this study including mostly SAH patients, we observed that the administration of inotropic agents was associated with an increase in brain oxygenation in 63% of patients; however, only half of those experienced a significant PbtO_2_ increase from baseline. The response to inotropic agents was greater in patients with lower PbtO_2_ values at baseline. There was a positive correlation between ΔPbtO_2_ and ΔCO and a negative correlation between ΔPbtO_2_ and ΔCPP.

The relationship between cardiac output and cerebral blood flow is complex and difficult to study in clinical practice (33). The use of inotropes to improve brain hemodynamics and oxygenation comes from the hypothesis of a so-called “cardio-cerebral coupling” (34, 35), where an increase in cardiac output could promote a proportional increase in CBF, in particular in case of impaired autoregulation and ischemia (36), both being common features of acute brain injury (37). We observed a positive correlation of PbtO_2_ changes with CO changes, suggesting the higher was the effects on global hemodynamics, the highest the changes in brain oxygenation. Also, CPP was reduced by the administration of dobutamine, probably due to its vasodilatory via β_2_ receptors, but this did not result in impaired cerebral hemodynamics. This might suggest the importance of systemic hemodynamics monitoring to adequately understand the effects of increasing CO on brain oxygenation (38) and the relevance of an “hyperdynamic” approach, rather than “hypertensive” one, to manipulate cerebral hemodynamics in ABI patients.

Previous studies conducted in SAH patients found that fluid boluses to improve CO yielded a positive correlation between changes in cardiac output and PbtO_2_ (39–41); to the best of our knowledge, this is the first study to assess the impact of inotropic agents on brain oxygenation in ABI patients. In experimental model of SAH, administration of dobutamine increased global CBF, which was initially reduced because of an induced brain injury, and resulted into a lower prevalence of DCI and better functional outcomes (42). Moreover, dobutamine may provide, through its effects on □2-receptors, some vasodilatory effects and protect vascular smooth cell muscle against the occurrence of vasospasm in an *in vitro* study (43). Furthermore, adding dobutamine and nimodipine to the management of cerebral vasospasm in SAH patients compared to nimodipine alone tended to improve the outcome of these patients (43).

Similarly, a study by Levy et al. found that dobutamine administration was able to clinically reverse vasospasm in 78% of patients, together with a significant increase in cardiac output (44). Also, in SAH patients with cerebral vasospasm, dobutamine effectively and significantly increased CBF, measured with Xenon-CT scan, independently from changes in blood pressure and similarly to norepinephrine (45). As for dobutamine, intravenous milrinone can also improve cardiac function after SAH (46) and reverse symptoms due to clinical deterioration caused by DCI in most cases (47), although no data on cerebral oxygenation are available.

It has been suggested that cerebral hemodynamics after an acute brain injury could be assessed using non-invasive monitoring, such as cerebral blood flow velocities or cerebral autoregulation indices. Nevertheless, cerebral autoregulation or CBF velocities cannot evaluate the adequacy of tissue oxygenation. As CPP target is routinely based on PbtO_2_ or other monitoring tools using vasopressors, our findings suggest that tissue hypoxia can be present in some ABI patients in the absence of elevated ICP and that systemic targets, including CPP or CO, should be individualized according to cerebral demand (15). As inotropic agents are not superior to norepinephrine alone to improve outcome in ABI patients at risk of cerebral ischemia (48), our results suggest that inotropic agents could be considered as a “second-line” therapy to improve brain oxygenation when initial CPP optimization with vasopressors has failed. Interestingly, the more significant effects on tissue oxygenation were observed in those patients with baseline tissue hypoxia, as reported for CPP augmentation or red blood cells transfusions (15, 17, 39).

This study has several limitations. First, this study has an important selection bias since the decision to start inotropes was based on the attending physician' opinion in the absence of an established protocol and due to its retrospective nature we could not determine what were the criteria used. Moreover, we included a very small number of patients, since inotropic treatment were used only in selected cases; therefore, this study is only hypothesis generating. Second, this is a single center study, which limits the generalization of our results. Third, we had only one patient who received milrinone and no comparison between the effects of different inotropes was possible. Additionally, milrinone was used in a lower dose than usually recommended for the treatment of refractory DCI (bolus of 0.1–0.2 mg/kg and continuous infusion of 0.75 mcg/kg/min up to 2.5 mcg/kg/min) (49, 50); however, in the case included in our cohort, this dose was sufficient to increase CO and improve PbtO_2_. Forth, we did not assess the clinical impact of inotropic administration, only its physiological effects on brain oxygenation; whether optimizing PbtO_2_ in TBI patients improves outcome remains to be demonstrated. Fifth, we did not take into consideration the exact positioning of the probes, i.e., some of those might have been placed into normally appearing areas, where no potential benefits from hemodynamic manipulation would have been expected. Sixth, we could not assess the correlation between cardiac output and PbtO_2_ changes in the subgroup analysis because of several missing CO values. Seventh, responders required higher doses of epinephrine after the start of inotropic treatment; therefore, we cannot exclude that the improvement in PbtO_2_ was due to the increased dose of vasopressors in addition to inotropic agents. Similarly, there was a slight increase in CO_2_ concomitant to the inotropic administration, which can also induce vasodilation and acts as important confounder. Finally, most of patients had SAH; whether this strategy is effective also in other forms of acute brain injuries remains to be further studied.

## Conclusions

In this study, the administration of inotropic agents could increase brain tissue oxygenation in nearly 65% of patients; however, only half of them had a significant PbtO_2_ increase. Baseline tissue hypoxia could predict this significant PbtO_2_ increase after the initiation of therapy. The effects on brain oxygenation were proportional to changes in cardiac output. Further prospective studies are needed to explore the impact of inotropic therapy on brain oxygenation and outcome in this setting.

## Data availability statement

The original contributions presented in the study are included in the article/supplementary material, further inquiries can be directed to the corresponding author/s.

## Ethics statement

The studies involving human participants were reviewed and approved by Erasme Hospital Ethics Committee. The Ethics Committee waived the requirement of written informed consent for participation.

## Author contributions

ED, GC, EG, and FT contributed to conception and design of the study and wrote the first draft of the manuscript. GC, AD, EM, and ED organized the database. EG performed the statistical analysis. SS, EM, AD, and JC revised the manuscript for intellectual content and English editing. All authors contributed to manuscript revision, read, and approved the submitted version.

## Conflict of interest

The authors declare that the research was conducted in the absence of any commercial or financial relationships that could be construed as a potential conflict of interest.

## Publisher's note

All claims expressed in this article are solely those of the authors and do not necessarily represent those of their affiliated organizations, or those of the publisher, the editors and the reviewers. Any product that may be evaluated in this article, or claim that may be made by its manufacturer, is not guaranteed or endorsed by the publisher.
