# Supplementary material for: Effect of inotropic agents on oxygenation and cerebral perfusion in acute brain injury
Source: Front Neurol. 2022 Jul 19;13:963562. doi: 10.3389/fneur.2022.963562 (PMC9343780; doi:10.3389/fneur.2022.963562)
Supplement: Supplementary file 1 [file Data_Sheet_1.pdf]

Supplementary Material

Supplemental Table S1: Characteristics of the study population. Data are presented as count (%) or median (IQRs).

**Variables**	**All patients (N=35)**
Age, years - median (IQR)	55 (46-62)
Male gender, n (%)	18 (51)
APACHE score on admission – median (IQR)	19 (12-23)
Glasgow Coma Scale on admission- median (IQR)	9 (3-13)
SOFA score at ICU admission - median (IQR)	9 (4-10)
Underlying disease, n (%)	
Non-traumatic SAH	31 (89)
TBI	2 (6)
ICH	2 (6)
**COMORBIDITIES**	
Alcohol abuse, n (%)	7 (20)
Smoking, n (%)	8 (23)
Drug abuse, n (%)	1 (3)
Arterial Hypertension, n (%)	14 (40)
Chronic Heart Disease (NYHA 3 or 4), n (%)	3 (9)
Diabetes mellitus, n (%)	3 (9)
COPD, n (%)	2 (6)
Liver Cirrhosis, n (%)	1 (3)
Cancer, n (%)	0
Previous neurological disease, n (%)	2 (6)
**DURING ICU STAY**	
Mechanical ventilation, n (%)	34 (97)
Days on mechanical ventilation, median (IQR)	10 (5-19)
Vasopressors, n (%)	34 (97)
RRT, n (%)	1 (3)
Sedation, n (%)	34 (97)
Opioid administration, n (%)	34 (97)
Anti-epileptics prophylaxis n (%)	34 (97)
**NEUROLOGICAL COMPLICATIONS**	
Intracranial hypertension, n (%)	21 (60)
Seizures, n (%)	7 (20)

Rebleeding, n (%)	3 (9)
Hydrocephalus, n (%)	17 (49)
Delayed cerebral ischemia, n (%)	19 (54)
At least one episode of brain tissue hypoxia, n (%)	32 (91)
**OUTCOMES**	
ICU LOS, days – median (IQR)	19 (13-26)
Hospital LOS, days – median (IQR)	26 (14-44)
ICU death, (%)	20 (57)
Hospital death, n (%)	20 (57)
GOS at 6 months – median (IQR)	1 (1-4)
Unfavorable neurological outcome at 6 months, n (%)	25 (71)

IQR: interquartile range; GCS: Glasgow coma scale; SAPS: simplified acute physiology score; NYHA: New York heart association COPD: chronic obstructive pulmonary disease; RRT: renal replacement therapy; ICU: intensive care unit; LOS: length of stay; GOS: Glasgow outcome score; SAH: subarachnoid hemorrhage; TBI: traumatic brain injury; ICH: intracerebral hemorrhage.

Supplemental Table S2: Comparison between responder and non-responder groups.

Variables	Non-Responders (N=23)	Responders (N=12)	p-value
Age, years - median (IQR)	55 (46-61)	56 (48-64)	0.48
Male gender, n (%)	12 (52)	6 (50)	0.54
APACHE score on admission – median (IQR)	19 (12-23)	20 (15-22)	0.85
Glasgow Coma Scale on admission- median (IQR)	5 (3-11)	5 (3-14)	0.72
SOFA score at ICU admission - median (IQR)	9 (5-10)	7 (5-9)	0.12
Underlying disease, n (%)			0.69
Non-traumatic SAH	20 (87)	11 (92)	
TBI	2 (9)	0	
ICH	1 (5)	1 (5)	
mFisher 3/4	19/20 (95)	10/11 (91)	0.99
WFNS 4/5	16/20 (80)	7/11 (64)	0.41
**COMORBIDITIES**			
Alcohol abuse, n (%)	6 (26)	1 (8)	0.38
Smoking, n (%)	6 (26)	2 (17)	0.69
Drug abuse, n (%)	0 (0)	1 (8)	0.34
Arterial Hypertension, n (%)	12 (52)	2 (17)	0.07
Chronic Heart Disease (NYHA 3 or 4), n (%)	3 (13)	0	0.54
Diabetes mellitus, n (%)	3 (13)	0	0.54
COPD, n (%)	1 (4)	1 (8)	0.99
Liver Cirrhosis, n (%)	0	1 (8)	0.34
Cancer, n (%)	0	0	-
Previous neurological disease, n (%)	1 (4)	1 (8)	0.99
**DURING ICU STAY**			
Mechanical ventilation, n (%)	23 (100)	11 (92)	0.34
Vasopressors, n (%)	23 (100)	11 (92)	0.34
RRT, n (%)	1 (4)	0	0.99
Sedation, n (%)	22 (96)	11 (92)	0.99
Opioid administration, n (%)	23 (100)	11 (92)	0.34
Anti-epileptics prophylaxis n (%)	15 (65)	10 (83)	0.43
**NEUROLOGIC COMPLICATIONS**			
Seizures, n (%)	2 (9)	5 (42)	0.03
Rebleeding, n (%)	0	3 (25)	0.03
Hydrocephalus, n (%)	12 (52)	5 (42)	0.73
DCI, n (%)	13 (57)	6 (50)	0.74
Intracranial hypertension, n (%)	12 (52)	9 (75)	0.28
At least one episode of brain tissue hypoxia, n (%)	22 (96)	10 (83)	0.27
**BASELINE VARIABLES**			
$PbtO_2$, mmHg	22 (19-28)	12 (8-16)	0.001
Hb, g/dL	11.5 (9.1-12.6)	10.9 (10-12.2)	0.69

MAP, mmHg	122 (117-132)	109 (88-120)	0.07
CPP, mmHg	115 (104-118)	94 (72-104)	0.03
ICP, mmHg	12 (6-14)	15 (13-20)	0.01
HR, bpm	85 (76-100)	94 (77-108)	0.50
Temperature, °C	37.0 (36.0-37.4)	36.7 (35.6-37.7)	0.88
pH	7.41 (7.37-7.46)	7.41 (7.36-7.43)	0.48
PaO_2, mmHg	109 (95-130)	112 (90-205)	0.67
$PaCO_2$, mmHg	44 (41-48)	39 (35-44)	0.06
Lactate, mmol/L	1.1 (0.8-1.4)	1.1 (0.9-1.2)	0.88
SaO_2, %	99 (98-99)	99 (97-100)	0.55
Glucose, mg/dL	135 (125-175)	130 (117-155)	0.77
SvO_2, %*	80 (79-82)	78 (73-83)	0.76
CO, L/min*	6.1 (5.1-8.2)	5.9 (3.5-7.4)	0.72
Norepinephrine, mcg/kg/min	0.43 (0.17-1.2)	0.35 (0.1-1.8)	0.80
**OUTCOMES**			
ICU LOS, days – median (IQR)	21 (16-28)	13 (8-18)	0.02
Hospital LOS, days – median (IQR)	31 (17-44)	14 (8-27)	0.09
ICU death, (%)	11 (49)	9 (75)	0.16
Hospital death, n (%)	11 (49)	9 (75)	0.16
GOS at 6 months – median (IQR)	3 (1-4)	1 (1-2)	0.31
Unfavorable neurological outcome at 6 months , n (%)	15 (65)	9 (75)	0.99

*IQR: interquartile range; GCS: Glasgow coma scale; SAPS: simplified acute physiology score; NYHA: New York heart association COPD: chronic obstructive pulmonary disease; RRT: renal replacement therapy; ICU: intensive care unit; LOS: length of stay; GOS: Glasgow outcome score; SAH: subarachnoid hemorrhage; TBI: traumatic brain injury; ICH: intracerebral hemorrhage. *n=25 (n=7 for responders and n=18 for non-responders).*

Supplemental Table S3 : Comparison of physiological variable before, one and two hours after inotrope administration according to type of response (responder and non-responder groups).

Variables	Responders			Non-responders			P value
	**Baseline**	**1h**	**2h**	**Baseline**	**1h**	**2h**	
$PbtO_2$, mmHg	12 (8-16)	16 (12-20)	19 (14-24)	22 (19-28)	20 (17-26)	20 (18-25)	0.001
Hb, g/dL	10.9 (10-12.2)	10.5 (9.8-12.4)	10.3 (9.9-12.1)	11.5 (9.1-12.6)	10.4, (9.2-11.9)	10.2 (9.2-11.8)	0.96
MAP, mmHg	109 (88-120)	106 (90-121)	99 (94-118)	122 (117-132)	127 (118-133)	122 (117-132)	0.85
CPP, mmHg	94 (72-104)	94 (68-109)	87 (75-109)	115 (104-118)	115 (107-121)	113 (103-122)	0.74
ICP, mmHg	15 (13-20)	15 (11-20)	13 (9-17)	12 (6-14)	9 (8-19)	9 (7-17)	0.06
HR, bpm	94 (77-108)	94 (85-104)	106 (86-109)	85 (76-100)	90 (79-107)	103 (90-109)	0.71
Temperature, °C	36.7, (35.6-37.7)	36.9 (35.7-38.8)	36.7 (35-37.8)	37.0 (36-37.4)	36.9 (35.7-37.2)	37 (35.9-37.3)	0.71
pH	7.41 (7.36-7.43)	7.42 (7.33-7.45)	7.41 (7.37-7.43)	7.41 (7.37-7.46)	7.43 (7.39-7.45)	7.42 (7.37-7.46)	0.23
PaO_2, mmHg	112 (90-205)	96 (75-111)	113 (98-126)	109 (95-130)	112, (101-140)	109 (93-138)	0.24
$PaCO_2$, mmHg	39 (35-44)	38 (33-44)	40 (35-46)	44 (41-48)	44 (39-46)	44 (39-49)	0.89
Lactate, mmol/L	1.1 (0.9-1.2)	1.4 (1-1.9)	1.3 (1.1-2.3)	1.1 (0.8-1.4)	1.1 (0.8-1.6)	1.1 (0.7-1.6)	0.52
SaO_2, %	99 (97-100)	98 (97-99)	99 (98-99)	99 (98-99)	99 (99-99)	99 (98-99)	0.32
Glucose, mg/dL	130 (117-155)	184 (133-233)	163 (139-285)	135 (125-175)	143 (113-179)	163 (125-190)	0.18
SvO_2, %*	78 (73-83)	79 (77-80)	79 (77-81)	80 (79-82)	78 (71-81)	85 (84-90)	0.36
CO, L/min*	5.9 (3.5-7.4)	6.4 (5.3-7.6)	6.8 (5.8-7.5)	6.1 (5.1-8.2)	7.2 (6.1-8.4)	7.9 (6.8-8.6)	0.78
Norepinephrine, mcg/kg*min	0.35 (0.1-1.8)	0.97 (0.18-2.4)	0.97 (0.3-2.2)	0.43 (0.17-1.2)	0.43 (0.14-1.4)	0.32 (0.08-1.17)	0.01
Dobutamine, mcg/kg*min	0	3 (2-5)	3 (3-5)	0	5 (3-5)	5 (3-5)	0.85

*$PbtO_2$: brain tissue oxygenation partial pressure; Hb: hemoglobin; MAP: mean arterial pressure; CPP: cerebral perfusion pressure mmHg; ICP: intracranial pressure; HR: heart rate; bpm: beats per minute. SaO_2: Oxygen saturation; PaO_2: Oxygen partial pressure; $PaCO_2$: Carbon dioxide partial pressure. *n=25 (n=7 for responders and n=18 for non-responders).*

Supplemental Table S4: Logistic regression model to identify factors associated with a significant $PbtO_2$ increase after inotrope administration in SAH patients (n=31)

	**Univariable OR (95% CI)**	**Multivariable OR (95% CI)**	**P value**
Baseline $PbtO_2$, mmHg	0.78 (0.65-0.95)	0.78 (0.64-0.95)	0.02
WFNS 4 or 5, mmHg	0.44 (0.08-2.27)	0.42 (0.04-4.16)	0.46
mFisher 3 or 4, mmHg	0.53 (0.03-9.34)	2.87 (0.05-153.46)	0.60

$PbtO_2$: brain tissue oxygen partial pressure; WFNS: world federation of neurological surgeons; mFisher: modified Fisher scale

.

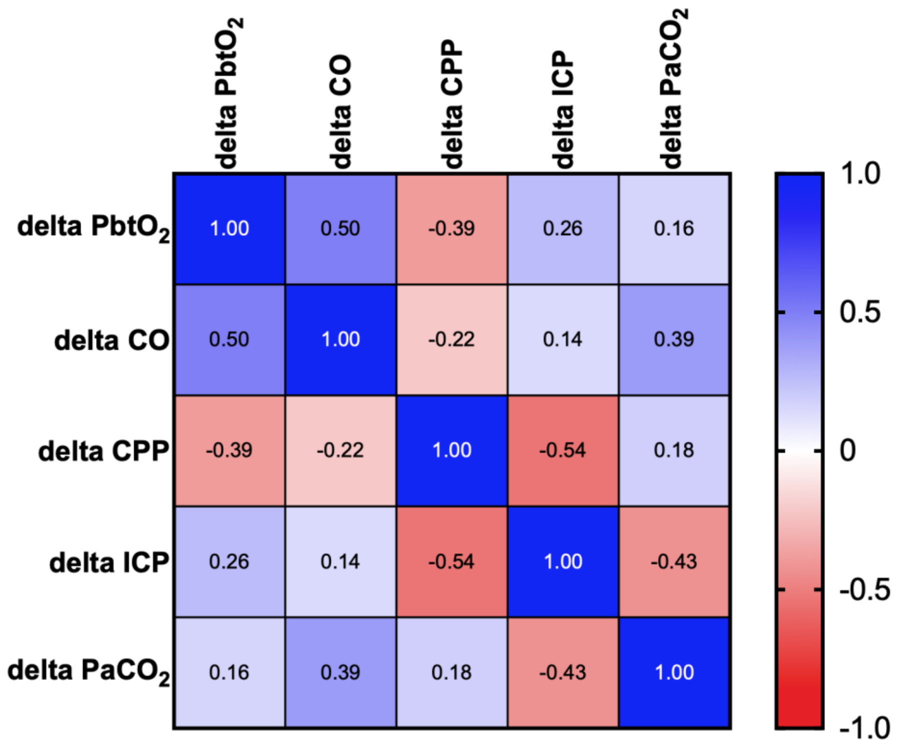

Supplementary Figure 1. Correlation matrix of $\Delta PbtO_2$ and ΔCO, ΔCPP, ΔICP and $\Delta CPaCO_2$ in the overall study population (CO, n=25).
